# Syndrome de blépharophimosis: une forme particulière du ptosis congénital

**DOI:** 10.11604/pamj.2015.20.282.5584

**Published:** 2015-03-23

**Authors:** Hanan Handor, Zouheir Hafidi, Mina Laghmari, Ihsane Sabrane, Olive Rosine Mastanga, Rajae Daoudi

**Affiliations:** 1 Université Mohammed V Souissi, Service d'Ophtalmologie A de l'Hôpital des Spécialités, Centre Hospitalier Universitaire, Rabat, Maroc

**Keywords:** Syndrome de blépharophimosis, ptosis congénital, anomalies palpébrales, Blepharophimosis syndrome, congenital ptosis, eyelid abnormalities

## Abstract

Le syndrome de blépharophimosis est une malformation palpébrale congénitale caractérisée par l'association d'un ptosis majeur bilatéral à d'autres anomalies palpébrales. Il constitue une forme particulière du ptosis congénital qui doit être connue par tout ophtalmologue afin d'optimiser la prise en charge des patients présentant cette affection.

## Introduction

Le syndrome de blépharophimosis est une malformation palpébrale congénitale caractérisée par l'association d'un ptosis majeur bilatéral à d'autres anomalies palpébrales. Il constitue une forme particulière du ptosis congénital qui doit être connue par tout ophtalmologue. A travers le cas clinique que nous présentons, nous décrivons un cas typique de ce syndrome.

## Patient et observation

**Figure 1:**
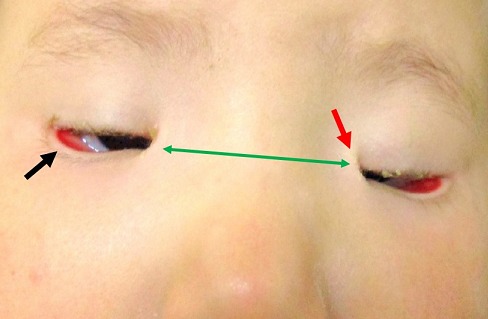
Photographie couleur de la face illustrant le ptosis majeur bilatéral. A) le télécanthus; B) l’épicanthus inversus; C) l'ectropion de la paupière inférieure

L'enfant L, âgée de 3 ans, a été adressée par un pédiatre pour un avis ophtalmologique devant la constatation d'un ptosis bilatéral. L'examen clinique retrouve un ptosis majeur bilatéral associé à un télécanthus (Figure 1), un épicanthus inversus (Figure 1) un ectropion de la paupière inférieure (Figure 1) et une absence du pli palpébral supérieur. La fonction des deux releveurs des paupières supérieures est nulle et l'enfant soulève les sourcils et adopte une attitude vicieuse de la tête (tête rejetée en en arrière) lors des efforts de fixation. Devant ce tableau clinique, le diagnostic de syndrome de blépharophimosis est posé. L'enfant présente par ailleurs, une esotropie avec une limitation de l'abduction au niveau des deux yeux. Aucune autre anomalie associée n'est retrouvée à l'examen à la lampe à fente.

## Discussion

Le syndrome de blépharophimosis est une malformation palpébrale congénitale, souvent de transmission héréditaire autosomique dominante [1]. Il se caractérise par l'association d'un ptosis bilatéral symétrique (souvent majeur), un télecanthus, un épicanthus inversus [2], une absence du pli palpébral supérieur, une brièveté du tarse et un ectropion de la partie externe de la paupière inférieure [3]. Pour compenser le ptosis, les patients adoptent des postures vicieuses permettant de dégager leurs axes visuels: hyper extension du cou et contraction du muscle frontal pour soulever les sourcils. Deux types de syndrome de blépharophimosis sont décrits: Le type 1, caractérisé par l'association d'une insuffisance ovarienne aux anomalies palpébrales suscitées. Le type 2, où les anomalies palpébrales sont isolées [4]. Ce syndrome peut aussi s'associer à d'autres atteintes ophtalmologiques à type de: microphtalmie, strabisme et colobomes papillaires [5]. L'amblyopie est souvent retrouvée au cours de ce syndrome, et semble être multifactorielle: ptosis majeur, strabisme, amétropies [6].

## Conclusion

A travers cette observation nous décrivons un cas typique du syndrome de blépharophimosis qui représente une forme clinique du ptosis congénital.Nous rapportons également les principales malformations qui s'y associent.
